# 738 Does A History of Malignancy Lead to Worse Outcomes in Burns?

**DOI:** 10.1093/jbcr/irac012.291

**Published:** 2022-03-23

**Authors:** Sanja Sljivic, Lori Chrisco, Rabia Nizamani, Booker King, Felicia Williams

**Affiliations:** University of North Carolina - Chapel Hill, Chapel Hill, North Carolina; North Carolina Jaycee Burn Center, Chapel Hill, North Carolina; UNC Jaycee Burn Center, Chapel Hill, North Carolina; UNC Jaycee Burn Center, Chapel Hill, North Carolina; UNC Jaycee Burn Center, Chapel Hill, North Carolina

## Abstract

**Introduction:**

A history of malignancy is associated with worse outcomes in cardiac disease and trauma. Our objective was to determine if a past medical history of cancer portends increased morbidity or mortality in burns or skin-sloughing disorders.

**Methods:**

Patients were identified using our institutional Burn Center registry and linked to the clinical and administrative data. All patients admitted between January 1, 2014 and June 30, 2021 were eligible for inclusion. Demographics, length of stay (LOS), co-morbid conditions, and mortality were evaluated. Statistical analysis was performed with Students’ t-test, chi-squared, and Fischer’s exact test.

**Results:**

A total of 8,018 patients were admitted during this period, and of those patients, 436 had a history of cancer (5%). Patients with a history of cancer were older (56 years versus 44 years), p< 0.0001. They had a significantly longer LOS. They had larger burns and higher hospital costs. They were more likely to be female and more likely to have a skin-sloughing disorder. Patients with a history of cancer also had higher mortality rates.

**Conclusions:**

A history of cancer is associated with worse outcomes in patients admitted for burn injury or skin-sloughing disorders. Further study is warranted to investigate and mitigate what aspect of their care could be adjusted to improve outcomes.

